# Strategies for engaging “hard-to-reach” populations in a panel for digital health research: A qualitative study among experts

**DOI:** 10.1371/journal.pdig.0001033

**Published:** 2025-10-09

**Authors:** Corine Oldhoff-Nuijsink, Mirjam P. Fransen, Linda W. Peute, Marloes E. Derksen

**Affiliations:** 1 Amsterdam UMC, location University of Amsterdam, Department of Medical Informatics, eHealth Living & Learning Lab Amsterdam, Amsterdam, The Netherlands; 2 Amsterdam Public Health research institute, Digital Health, Amsterdam, The Netherlands; 3 Amsterdam UMC, location University of Amsterdam, Department of Public and Occupational Health, Amsterdam, The Netherlands; 4 Amsterdam Public Health research institute, Quality of Care, Amsterdam, The Netherlands; 5 National Institute for Public Health and the Environment, Centre for Prevention, Lifestyle and Health, Department of Behavior and Health, Bilthoven, The Netherlands; The University of Sheffield, UNITED KINGDOM OF GREAT BRITAIN AND NORTHERN IRELAND

## Abstract

Digital health technologies are developed to aid individuals in managing their health. Nonetheless, a significant number of these technologies remain neither implemented nor utilized by potential end users. One contributing factor to this gap in uptake is the insufficient consideration of the target audience needs and requirements during the development phase of these technologies. Moreover, certain groups in society are often underrepresented in such research projects (so called “hard-to-reach”), leading to a disconnect between the developed technologies and their needs and requirements. However, recruiting a representative study population – including individuals from different demographic backgrounds - for such studies poses challenges for researchers. One proposed solution is panel research, wherein a fixed group of participants is willing to participate in multiple research projects over time. In this study, we conducted semi-structured interviews with twelve experts in panel management or with researchers working with individuals in a vulnerable position, to gain insights into their experiences. Through thematic analysis, four key themes emerged: diverse recruitment strategies, investment in sustainable participation, simplified informed consent, and regulating practical matters. Recruiting a representative study population requires diverse and active strategies, such as visiting community centres and leveraging key figures. Long-term engagement can be maintained through regular, accessible communication, flexible participation options, and aligning research goals with participants' interests. Additionally, clear expectations, a supportive environment, respect for privacy, and feedback and incentives are crucial for retaining panel members. Taken into account these factors support inclusiveness in digital health research. Ultimately resulting in better alignment between users’ needs and the development, implementation and adoption of digital health technologies.

## Author summary

In today's world, many digital technologies are being created to help people manage their health. However, these technologies are often unused because they do not always meet the needs of the people that require most support. One reason for this is that individuals who struggle with digital technology are often left out of the research process, because it takes researchers a lot of time and effort, and researchers perceive it as complex to engage these groups. Underrepresentation, of people having difficulties accessing and using digital health technologies, can lead to a mismatch between what is developed and what is actually useful. In our study, we explored how researchers can better include these individuals by speaking with experts who manage long-term research panels and researchers who work with vulnerable groups. We found that successful inclusion and retention depends on using a variety of outreach methods, building long-term relationships with participants, simplifying the consent process, and handling practical issues with care. By making research more inclusive and responsive to people’s real needs, we can create digital technologies that are more effective and widely used.

## Introduction

The development of digital health technologies opens up numerous opportunities for enhancing healthcare, for example by expanding outreach and accessibility to underserved populations [1,2]. Digital health can help bridge disparities by increasing access and participation for individuals who might not be able to attend in-person programs. This includes people living in rural areas, those with caregiving duties or transportation challenges, individuals with irregular work hours, persons with disabilities, and those from lower socioeconomic backgrounds [3]. Additionally, these technologies are promising in reaching and informing individuals with lower health literacy by tailoring information or language based on user preferences [4,5]. However, studies examining the outreach, engagement, and effectiveness of digital health reveal that these technologies do not benefit everyone equally [6–8]. In fact, they may even widen the gap in health equities [6,9,10], either disparities in health conditions or the allocation of health care resources among different populations [11,12].

Numerous digital health technologies fall short of adequately addressing the needs and requirements of (potential) users [13,14]. Consequently, these technologies are neither implemented nor utilized by the intended end users, thereby failing to achieve their intended purposes. The discrepancy between digital health technologies and user requirements can be attributed, in part to the absence of a representative study population during the development and evaluation phases of these technologies [15]. This underrepresentation is particularly present in groups that are “hard-to-reach” for researchers, as they are not reached with traditional recruitment methodologies [16,17]. It appears to be challenging and time-consuming for researchers to recruit representative individuals [16–18]. These so called “Hard-to-reach” groups cannot be defined unequivocally, they may include disabled individuals, elderly, homeless people, refugees, individuals with mental health problems, minority ethnic groups, those who live in relative rural isolation, or individuals with low (digital) health literacy [19–24]. Consequently, this underrepresentation limits the generalizability of findings of digital health technology research [3].

To promote inclusive research and the development of accessible and useful digital health technologies, the engagement of a representative group should be a fundamental component of digital health research [19]. Including a diverse population is essential to ensure that the needs and requirements of all potential end users are represented in the design of the technologies, in accordance to the user centered design principles [25]. User centered design is an iterative design process in which designers concentrate on the users and their requirements at each phase of the design process.

A possible solution, for the utilization of a representative study population, could be the establishment of a research panel. A research panel consists of a diverse group of individuals, who participate in multiple digital health development, implantation or evaluation studies. One of the primary advantages of utilizing a panel design is that participants do not need to be re-recruited for each successive study, we expect this to enhance efficiency and reduce sampling costs. Earlier research by Fairbrother et al. who established a clinical patient panel for home telemonitoring showed potential [26]. They reflected on the panel as a culture of openness and respect that fostered trust and collaboration between panel members and researchers. Which led to productive meetings and growing confidence in involving patients in research development [26]. However, literature lacks relevant information on the formation, establishment and sustained maintenance of a research panel with a representative study population – including “hard-to-reach” individuals - for digital health research.

This study therefore answers the question*: How can we stimulate the engagement of representative individuals for the development, implementation, and evaluation of digital health technologies, according to domain experts?*

## Methods

In this observational study, semi-structured qualitative interviews were conducted among domain experts, in the Netherlands. We followed the COREQ criteria for reporting this study [27]. The Medical Ethics Review Committee of Amsterdam UMC agreed upon this study as non-medical scientific research (2023.0289).

### Participants and recruitment

We applied purposive sampling, we searched on the Internet for relevant Dutch research panels, advisory groups conducting health related research and researchers conducting research with individuals in a vulnerable position. In December 2023, we contacted 10 panels/ advisory groups and 2 researchers by e-mail. Neither the number of panelists nor the inclusion of qualitative alongside quantitative methods was considered a inclusion criteria. However, we excluded panels that primarily focused on commercial research.

Prior to participation, experts were provided with an information letter outlining the aims of the study. They were assured that all collected data would be pseudonymized. Written informed consent was obtained prior to the interviews.

### Data collection

The interviews were conducted by two researchers (CON [Junior researcher, female, MSc in Health Sciences, experienced in interviewing] and MC [Junior researcher, female, MSc in Medicine, got interview training prior to interviews]), in January 2024. Additional interviews were conducted to achieve data saturation, in August 2024. The interviews were one off. Interview questions were pilot tested with a colleague for understanding of the questions, length of the interview and if relevant questions were missing. The interviews took place either via MS Teams or in person at experts’ workplaces. Researchers had no relationship established with participants prior to the study. During the interview, no one else was present besides the participant and the researchers. All interviews were in Dutch and lasted approximately 45 minutes. Following the interview, the experts were compensated with a €20,- gift card. The interviews were audio recorded and notes were made during the interview.

The interview guide was based on the Business Model Canvas [28] and on attributes of inclusive health research suggested by Frankema et al. [29]. This guide included questions on the establishment, strategy, operationalization, organization, legal affairs, financial matters, recruitment, challenges and solutions for inclusive research, and communication with panel members. Afterwards, participants were asked to complete a short questionnaire to collect baseline expert characteristics (i.e., organization, function, and years of experience).

### Data analysis

The raw interview data was transcribed using the transcription function in MS Word. MC manually enhanced the computer transcripts so that the audio recording were transcribed verbatim. Transcripts were not send to participants for comments, as this was not consented to the participants on the informed consent form. Prior to coding the data, MC prepared a coding tree based on the interview questions (i.e., Business model Canvas and principles for inclusive research). Data was thematically analyzed using MAXQDA Plus 2022 [30].

In the initial coding round, MC and CON independently coded two interview transcripts. MC and CON held a consensus meeting to discuss discrepancies, most disagreements were about the length of the coded citations. Eventually, MC and CON agreed on all the coding. MC coded all remaining transcripts.

The data analysis process involved iterative steps. First, MC deductively coded the data using a predefined coding tree. Data analysis was complemented with an inductive approach: by constant comparison within and between interview transcripts. Newly identified codes were incorporated into the coding tree using an open coding technique. Previously coded interviews were reanalysed to identify the presence of these new codes.

Subsequently, the relationships between codes were (re)defined to generate core and subthemes, utilizing axial and selective coding principles. The final themes were chosen based on the number of citations.

Authors agreed that data saturation was reached, when no new themes emerged from the last two transcripts. Following data analysis, MC translated Dutch quotations from the transcripts into English, which were double-checked by CON. Participants did not provide feedback on the findings, as this was not consented to the participants on the information consent form.

## Results

We interviewed 12 experts. Seven were experts in panel management operating in large panels (>1.000 panelists) primarily focused on questionnaire based research or cohort studies, however some of them also conducted qualitative research (i.e., focus groups or co-creation). Three experts were involved in smaller advisory groups (with +/- 15 panelists) especially focused on people in a vulnerable position, performing only qualitative research. Two were experts with experience conducting research with people in a vulnerable position (response: 100%). Most of them were female (n = 10). They worked at different organizations across the Netherlands and had different functions, including researchers (n = 4), coordinator data collection (n = 1), project coordinator (n = 4), strategic advisors (n = 1), research assistant (n = 1) and a panel director (n = 1). The average active time in this function was 7.7 years (range 0.5-25 years). Most of them (n = 11/12) worked at a non-profit (academic) institute, one expert was employed at a panel performing commercial as non-commercial research.

### Themes

**Fig 1:**
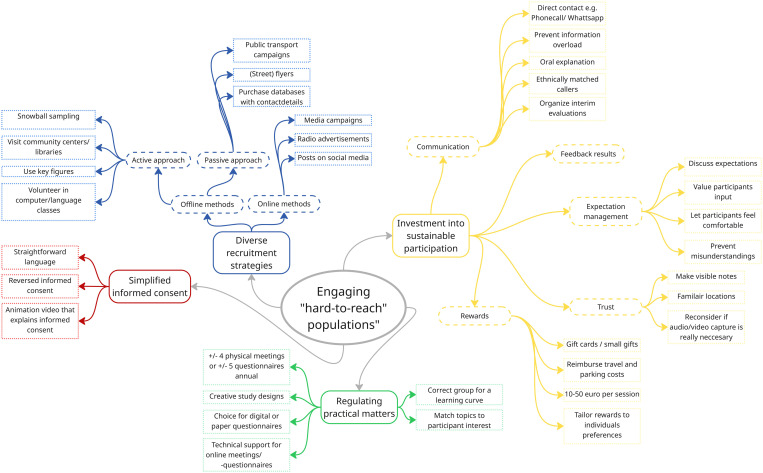
Strategies for engaging “hard-to-reach” populations in digital health technology research.

Data analysis resulted in four key themes, including (a) diverse recruitment strategies, (b) investment into sustainable participation, (c) simplified informed consent, and (d) regulating practical matters (see Fig 1).

### Diverse recruitment strategies

Experts explained that in various panels, a diverse array of recruitment methodologies was being employed to ensure a broad and representative participation across different demographics. Both online and offline methods were utilized. Online methods encompassed media campaigns, radio advertisements, and digital approaches such as posts on online platforms and social media (i.e., LinkedIn, Facebook).

The offline recruitment methods that experts described could be divided into passive and active approaches. Passive recruitment methods included public transport campaigns and distributing flyers with QR codes for panel registration. Lastly, some panels purchased databases with e-mail addresses to invite citizens for participation by e-mail. According to the experts, passive recruitment was especially suitable for panels with an open enrolment policy, meaning they allowed anyone to sign up through their website, mail, or phone. However, to still be able to maintain inclusion and exclusion criteria in their policy, experts recommended clearly stating eligibility on recruitment materials. For example, ‘*we are seeking individuals who struggle to make ends meet’.* Besides that, experts stressed that it is important to communicate on the flyer that the research is not an exam, but explicitly state that researchers are interested in the experiences and opinions of panellists. According to them, this would ensure that participants did not experience pressure, allowing them to feel comfortable.

Experts mentioned various active approaches to involve community engagement efforts such as visiting community centres or libraries, to engage with visitors. Some researchers volunteered in language or computer classes to build rapport. According to them, this approach enabled researchers, together with individuals, to identify challenges they faced in their daily lives. Subsequently, these challenges served as input for research projects, making participation more attractive to individuals. Experts frequently indicated that an active approach yielded more responses than a passive approach. Some panels collaborated with home care organizations, where nurses administered surveys during home visits. Moreover, collaborations with health centres (i.e., GP offices or physiotherapist), religious institutions (i.e., churches, mosques), educational institutions (i.e., libraries, vocational schools), and organizations focused on literacy and patient advocacy further enhanced outreach. Experts indicated that these locations often attracted individuals who were in a relaxed setting and generally enjoyed participating in activities, such as research studies.

*“Indeed think about where do these people in vulnerable positions come anyway, like the library or certain counters where they can ask for information about DigiD [secured log in method of the Dutch government] or something like that, so go there, I would say.”* Expert 9, small panel, 2 years work experience in this function

Experts also highlighted the effectiveness of leveraging personal networks and using snowball sampling. They explained that this approach stimulated confidentiality, and reduced suspicion towards researchers. As an example, one of the experts mentioned using key figures from migrant communities often served as cultural bridges, facilitating engagement..

*“Actually, at many organizations we visit, I often bring Fatiha, who has a Moroccan background, and Lorette, who has a Surinamese background and also a background from Curaçao. So, I do indeed try to bring language ambassadors with diverse backgrounds, but they must be reasonably proficient in speaking and understanding Dutch.”* Expert 7, small panel, 25 years work experience in this function

Two experts indicated that they did not involve the target population themselves but rather worked with representatives, (e.g., community centre staff or ex-low-literates). They argued that the representatives were easier to reach, and could better articulate what challenges these people faced.

*“It is also difficult for someone who is in the middle of a difficult situation to then reflect on certain things. Sometimes it’s just nice when someone is already a little bit in a better position, so they can reflect on how it was.”* Expert 9, small panel, 2 years work experience in this function

### Investment into sustainable participation

Experts emphasized the importance of fostering long-term engagement with panellists. Key facilitators included clear communication, feedback of results, expectation management, trust-building, and offering appropriate rewards.

### Communication

Most experts agreed that maintaining regular communication with participants was key to sustaining engagement in panel research. A few experts involved in smaller panels, used WhatsApp to communicate with panellists to ensure timely and direct interactions. Other experts communicated by phone to explain the information letter and answered any questions panellists’ had. They noted that some panel members found an information letter via email intimidating, as they did not understand what was important for them and experienced information overload.

Most experts used e-mail to send newsletters with updates about the panel. Others sent holiday and birthday cards by post, to help build a sense of community and appreciation. They mentioned that most panellists appreciated the newsletters and cards.

Interim evaluations helped identify what panel members appreciated and what they wanted to see done differently. Experts mentioned that taking this seriously helped to keep panel members motivated.

*“We asked the panel itself, ‘We’ve been at this for a while. How do you think it is going? How do you feel about the mutual involvement? What can we do better?’ The main feedback was that they find the content meetings where things are reported back to be very valuable.”* Expert 10, large panel, 3 years work experience in this function

### Feedback and expectation management

All experts mentioned that providing feedback to participants, about research they had participated in was crucial, preferably in the short term. Most panels sent this via e-mail. This approach not only kept participants informed but also showed them how their contributions were valued and utilized, strengthening their commitment to the research. This was also necessary for some panellists who experienced anxiety about not meeting expectations (fear of failure) or felt inadequate, fearing their contributions would not add value. Therefore, panels had to offer flexible participation options that accommodated various life circumstances to sustain their commitment.

*“Sometimes they just don’t feel well physically or mentally to participate, so they want to have the option to say no occasionally.”* Expert 9, small panel, 2 years work experience in this function

On the other hand, an expert mentioned that they received all kinds of questions from panel members, related to their health or health insurances. This cost the panel a lot of time and was not the intended purpose of the panel. Therefore, managing expectations beforehand was crucial to prevent misunderstanding and to avoid unnecessary investment of time in members who did not actively contribute to research projects.

### Trust

Building trust was a frequently mentioned topic among the experts with experience in conducting research with vulnerable groups. One experts explained that research in general seemed to lead to distrust among these groups. When the expert once asked a group of people from secondary vocational education what their associations with the word ‘research’ were, the responses were:

*“Well, words like police, justice, hospital, those were all associations that I would not first associate with research, but it was, that was in fact the living environment of these young people, because research? Yes, the police also performs research. If you have to deal with the police. So you also have to be careful with that word [research], because especially with young people that have a background as a refugee, yes, they have all been with the […] and they have all been researched.”* Expert 8, working with vulnerable groups, 3 years work experience in this function

To mitigate distrust among panellists when they expressed their opinions and experiences, one expert recommended making visible notes on a large tablecloth instead of using a laptop or audio recording the session. According to most experts, it was important to build trust right at the invitation. They suggested to presenting photos of researchers on the flyer in order to emphasize confidentiality. Additionally, holding meetings in accessible and familiar locations, such as community centres rather than hospitals, can help alleviate anxiety. All these factors helped to comfort panellists. As a result, they were more open during activities such as think-aloud sessions, which was crucial for gaining deeper insights into their cognitive processes.

### Rewards

Incentives could play a role in encouraging participation in panel research. According to experts, effective rewards include (the random drawing of) gift cards and distributing various vouchers, such as from supermarkets, web shops, or tourist offices. Some provided small gifts (e.g., chocolates or flower seeds) to participants after physical meetings. Experts differed on whether a reward was necessary- some giving no reward on the grounds that it should not become the main incentive for participation. Others did reward panellists to show appreciation for the time and energy participants invested. It was important to consider the needs of the target audience. Multiple experts considered it important to tailor the rewards to individual preferences, as explained in this quote:

*“Considering the context and situation of an individual, because, for example, we cannot offer anyone a permanent job. And if someone happens to be on welfare benefits and you pay them for a few hours, their benefits might be reduced, and they may not be able to regain them.”* Expert 8, working with vulnerable groups, 3 years work experience in this function

Another expert mentioned, that for individuals living in poverty, luxury gift cards were deemed less beneficial compared to cash or supermarket vouchers.

*“I think there is a site cadeaubonnen.nl [giftcards.com], you can then choose […]. All those vouchers are just not the right compensation. It’s just not the right compensation for this target group. They have no use for an ICI Paris or a Douglas [perfume company] voucher. “* Expert 3, working with people in poverty, 1 year work experience in this function

All experts indicated that travel expenses should always have been reimbursed, either through a financial form or by providing tickets for public transport. The amount of compensation ranged from 10 to 50 euros per session, depending on participants’ workload capacity (e.g., 50 euros for a two hour session). Some panels implemented a reward system where participants could choose a reward worth 10 euros after collecting a certain number of points.

Notably, some experts indicated that their panellists preferred feedback over monetary incentives. Approximately one-third of the participants declined gift cards. Additionally, some panellists raised concerns about privacy, particularly regarding university reimbursement forms that required extensive personal information, such as social security numbers, which many participants were hesitant to disclose.

### Simplified informed consent

Almost all experts ensured that written informed consent was obtained while registering members for the panel. Panels working with vulnerable populations specifically noted that obtaining written informed consent was challenging. Experts explained that participants often had difficulties reading or understanding the provided information.

*“If I presented it [informed consent letter] to someone, first of all he would not understand it. He would only sign it because I would tell him to sign it. Or I am not even telling him to sign it, I am explaining it to him correctly. But most people’s Dutch is too limited to read that. So yes, then he signs something that he does not understand, and how ethical is that?”* Expert 8, working with vulnerable groups, 3 years work experience in this function

Experts made suggestions on how to simplify the informed consent procedure. Creating an animation film to explain informed consent ensured clarity and understanding among participants. Using straightforward language was considered crucial, as it was unethical for participants to sign a consent form without fully comprehending what they were agreeing to. Reversed informed consent - where the participant initiated the consent process and the researchers agreed to respect and guarantee their privacy - and the use of illustrations further enhanced comprehension. This approach made the process more inclusive and accessible, ensuring all participants were truly informed before they provided their consent.

## Regulating practical matters

### Study design and frequency

According to experts, the appropriate implementation of research frequency was essential to maintain participant interest and ensure a continuous collection of data. Most experts indicated that surveys were conducted approximately five times a year, while physical meetings occurred up to four times annually. Some experts noted that traditional methods, such as interviews or focus groups, were not always the most effective for collecting data from vulnerable groups. Creative methods, which allowed participants to share their experiences in their own words rather than through structured questions, often resulted in richer data. For example, participatory action research, where individuals were actively involved and observed, often proved more effective.

*“I think I’ve actually been able to get the in-depth information we’ve obtained only by just going out and doing things instead of just talking. Because that’s all so verbal.”* Expert 8, working with vulnerable groups, 3 years work experience in this function

### (Technical) support

In the context of survey research, participants were given the option to choose their preferred method of completing questionnaires - whether digital (including text-to-speech features), paper (including free return envelopes), or telephone surveys. Completing surveys in their own environment often resulted in more thoughtful and considered responses, sometimes in consultation with their partners. Experts also recommended keeping questionnaires concise and ensuring they functioned smoothly on both computers and mobile devices to maintain participant engagement. Some panels offered support for logging into virtual meetings, such as MS Teams, particularly for individuals who struggled with digital tools. Additionally, providing a helpdesk to assist with any technical issues further supported participants.

Experts emphasized the importance of providing (technical) support, as it helped to keep individuals who struggled with, for example, digitalization engaged in research projects. This was considered crucial because the perspectives of these individuals were particularly valuable for a holistic research approach and supported research inclusiveness. For example, one expert mentioned that when they started sending paper questionnaires per post instead of only providing them by e-mail, the response rates increased. Their panellists noted that emails often ended up in spam folders.

### Learning curve

In order to organize a panel with a fixed percentage of participants who have low (digital) health literacy levels, and thus be representative, experts felt it was important to measure this regularly. If participants remained in a panel for an extended period, a learning curve could develop, causing them to no longer meet the inclusion criteria. Additionally, there were panels where members could only be part of the panel for a maximum period of five years, thereby correcting for a learning curve.

### Research climate

For physical meetings, experts noted the importance of maintaining an informal atmosphere, for example by providing some refreshments. An expert working with vulnerable groups stated that it was crucial to continually check in on what mattered to the target audience. Panellists were asked which topics were important to them, and together they brainstormed about potential research topics. It was also considered relevant not only to gather information from panellists but to ensure participants benefited from information they found interesting - for example, by organizing a speaker on an topic of interest to them (e.g., health insurance or healthy lifestyle). Furthermore, organizing public days also served to engage the community and foster a sense of involvement and commitment.

*“We also asked them, “What would you find beneficial?” And the response indicated that they would like to receive information from experts and researchers on various topics, such as lifestyle and health insurance. Indeed, a wide range of subjects were brought up.”* Expert 3, small panel, 1 year work experience in this function

## Discussion

### Main findings

This study explored how to recruit and engage a representative study population for the development, implementation and evaluation of digital health technologies, specially focussed on the so called “hard-to-reach” individuals, according to experts. One of the main findings is that recruitment strategies aimed at “hard-to-reach” individuals can benefit from effective collaboration with various entities, such as community centres or libraries where computer classes are offered. Another main finding is the importance of building trust, particularly in qualitative research methods such as interviews, co-creation sessions, or usability testing. Experts stated, trust can be fostered by expressing appreciation for participants’ contributions and by emphasizing that researchers are genuinely interested in their opinions and experiences, reassuring them that there are no incorrect answers or perspectives.

Another important finding is the need to address and alleviate any barriers or concerns that potential panellists may have. Employing ethically matched callers or researchers can help them feel more at ease, which in turn encourages them to express themselves more freely. Experts involved in this study also emphasized that interview questions should not be overly complex, lengthy, or contain multiple questions at once. Allowing panellists the space to speak freely is considered paramount. Furthermore, this study found that research involving panel members must be tailored to their needs, underscoring the importance of flexibility. Experts believed that panellists appreciate being involved in research that aligns with their interests, as it gives them a sense of relevance and personal connection to the study.

Expectation management emerged as another significant theme. Panellists need to understand what is expected of them and recognize their value to the study, at forehand. This clarity enables them to make informed decisions about their willingness and ability to participate. Additionally, several experts noted that panel members often do not fully understand the informed consent procedure and may be reluctant to admit this. Although panel members may sign the consent form, some researchers question the ethical implications of obtaining consent without full comprehension. To address this, some researchers choose to orally explain key sections of the form or simplify the document altogether. In certain cases, such as participatory action research where no personal data is collected, the consent form may be omitted entirely.

### Integration with prior work

No comparable studies were found that specifically address the involvement of a representative, nor “hard-to-reach” population in panel research. However, several studies have been published engaging “hard-to-reach” populations—such as older adults, individuals in socially vulnerable positions, or those with limited health literacy—in general health related research. The findings of our interview study are consistent with and build upon existing literature among these groups.

For instance, Rockliffe et al. [31] who investigated recruitment strategies among “hard-to-reach” groups for interview studies and Andersson [32] among Swedish outreach- and youth workers, both underscore the need for proactive recruitment and the cultivation of respectful relationships as foundation to successful engagement. These principles were echoed in our study, where participants responded positively to personalized outreach and culturally sensitive facilitation. The use of ethically matched researchers, was supported by prior work [33,34], they described the value of mutual understanding and ease of expression, reinforcing shared identity in bridging communication gaps.

Moreover, our findings align with Galvano’s behavioural analysis [35], which highlights the cognitive burden that complex or lengthy questions can impose – particularly on participants with limited literacy or cognitive capacity. This is further supported in a systematic review conducted by Liljas et al. [36]), who advocate for flexible formats, such as home visits can, to accommodate participants’ needs. Similarly, Davies et al. [37], described the need for flexibility in research design in a study among people aged 85 and over. Arguing that older adults may benefit from shorter, more frequent sessions and that flexibility in timing and structure can enhance participation.

Finally, the challenges surrounding informed consent are well-documented in the literature. Previous research has shown that individuals would benefit from simplified consent procedures [38]. Our findings reinforce the need for simplified, accessible consent procedures. As demonstrated by Garrett et al. [39], who found that simplified consent forms improved both comprehension and willingness to participate.

Taken together, these studies not only validate our findings but also highlight a shared set of principles – respect, flexibility, cultural sensitivity, and clarity – that are essential for engaging “hard-to-reach” populations meaningfully.

## Strengths and limitations

Strengths of the study include the engagement of experts in various positions, ranging from research assistants to panel directors. Some experts were involved with large panels comprising over 10,000 participants, while others managed small advisory groups of only ten individuals. This diversity provides a multidisciplinary perspective on the recruitment and engagement of panel members.

However, our study has some limitations. There is potential for subjectivity in data analysis and interpretation. To mitigate this, we involved a second coder in the data analysis process. Second, data were collected exclusively from Dutch experts. While this provides valuable insights grounded in the Dutch context it may limit the generalizability of findings to other national settings. The Dutch setting is characterized by strong institutional transparency, decentralized governance, and the relatively high trust in science. Most citizens have a mobile phone (91%) and the country also leads the EU in house hold internet access (98%), which exceeds the EU average 92% [40]. Additionally, it has well developed infrastructure for supporting research panels. In contrast, countries with more limited resources may face significant challenges in implementing similar methodologies. Furthermore, the proportion of individuals with excellent general health literacy in the Netherlands is high (25.1%) compared to other countries such as Austria (9.9%), Spain (9.1%) or Bulgaria (11.3%) [41]. Contextual features - like logistical arrangements, trust in science, and communication practices - are closely tied to Dutch cultural and policy context and therefore less easily generalizable. In contrast, other findings as discussed in the paragraph below ‘implications for research and practice’ are not inherently dependent on culture or policy frameworks and therefore more easily generalizable. Third, we employed purposive sampling and conducted an online search to identify relevant panels/ advisory groups, however it is possible that we overlooked pertinent panels that do not maintain an online presence.

## Implications for research and practice

The results of this study lead to implications for panel research practice, but are mostly also applicable to general health related research. The first implication is to adopt a participant-centered approach, particularly when engaging “hard-to-reach” populations in research and digital health development. To ensure inclusivity and sustained engagement, panel managers or researchers must employ strategies such as personalized invitations, culturally sensitive communication, and accessible meeting locations. The use of creative and participatory methods, alongside flexible data collection methods, enhances data richness. Regular assessment of panellists skills or literacy levels is essential to maintain representativeness, especially in long-term panels where learning effects may alter eligibility. Moreover, ethical considerations around informed consent require simplification through visual aids, plain language, and participant-initiated consent processes. Incentives, should be tailored to individual preferences and balanced with non-monetary forms of appreciation, such as feedback and community engagement. Technical support, including a helpdesk and assistant with digital tools, is vital to prevent exclusion.

All of the factors mentioned above can be taken into account when establishing a panel focused on the development, implementation and evaluation of digital health technologies. Further research could examine whether the panel has successfully included a higher proportion of “hard-to-reach” individuals, and whether these individuals where adequately represented across various research projects. A follow-up explorative study could examine the effects (e.g., alignment with user preferences and requirements) of involving diverse panellists on the development of digital health technologies. It could also assess whether the evaluation of these technologies (e.g., usability metrics) yields more comprehensive insights compared to digital health technology studies using traditional recruitment methods. Finally, further research should assess the scalability and adaptability of inclusive panel practices across different research contexts, to inform broader implementation in public health and social research.

## Conclusion

This study concludes that recruiting a representative study population necessitates the use of diverse recruitment strategies. Active strategies, such as visiting community centres and engaging key figures, seem particularly effective. To maintain long-term engagement of panellists, it seems essential to implement regular and accessible communication, offer flexible participation options, and continuously align research goals with participants’ interests. Additionally, providing clear expectations, fostering a supportive atmosphere, respecting privacy concerns, and acknowledging participants’ contributions through feedback and incentives are crucial for retaining panel members. All these strategies will help enhance inclusivity of digital health research. Ultimately leading to a better alignment between user requirements and digital health technologies.

## Supporting information

S1 File Questionnaire background characteristics. (PDF)

S2 File Interview guide. (PDF)

S3 File Information letter. (PDF)

S4 File Informed consent. (PDF)
