# Supplementary material for: Strategies for engaging “hard-to-reach” populations in a panel for digital health research: A qualitative study among experts
Source: PLOS Digit Health. 2025 Oct 9;4(10):e0001033. doi: 10.1371/journal.pdig.0001033 (PMC12510573; doi:10.1371/journal.pdig.0001033)
Supplement: S1 File — (PDF) [file pdig.0001033.s001.pdf]

Questionnaire

Please complete these questions after the interview with the researcher. This information helps to get a complete overview of your (work) situation. All information is processed anonymously and cannot be traced back to you.

1. In which year were you born?

   ____

2. What is your gender?

   ____

3. At which organization do you work?

   ____

4. What is your position in this organization?

   ____

5. How many years have you been working in this position?

   ____
