# Supplementary material for: Strategies for engaging “hard-to-reach” populations in a panel for digital health research: A qualitative study among experts
Source: PLOS Digit Health. 2025 Oct 9;4(10):e0001033. doi: 10.1371/journal.pdig.0001033 (PMC12510573; doi:10.1371/journal.pdig.0001033)
Supplement: S2 File — (PDF) [file pdig.0001033.s002.pdf]

# INTERVIEW GUIDE

The purpose of this interview is to learn more about the establishment and maintenance of a research panel. We hope to achieve this goal by understanding the experiences around existing panels, such as yours. The interview will take about 45 minutes.
[Do you have questions or remarks on forehand?]
[Do you agree with audio recording?] Start audio

## Interview questions

**General**

1. Can you tell me something about the panel you are coordinating/ working for?
2. In which year was the panel set up?
    - 2.1. For what purpose was the panel set up?
        - 2.1.1. Does the panel currently still meet these goals, or have the goals been adjusted over the years?
    - 2.2. How many members does the panel have?
        - 2.2.1. What percentage of these are active members and therefore always provide responses?
        - 2.2.2. Do you also select certain groups from the panel? E.g. only women/ certain age groups / vulnerable groups
    - 2.3. How many people were involved in setting up the panel?
        - 2.3.1. What were their tasks?

**Strategic**

3. With what (social) vision did you set up the panel? What was/is your mission/drivers?
    - 3.1. What type of studies do you use your panel for? (questionnaire studies, focus groups, interviews, etc.)What do you want to achieve with the panel in the future?
        - 3.1.1. What do you think are new developments/opportunities (digitalisation, AI etc.)? What do you want to capitalise on?
        - 3.1.2. What risks play a role when using your panel in research projects?

4. With which organisations do you collaborate?
    - 4.1. Can you elaborate on this?
    - 4.2. How did this relationship come about? (For whatever reason)
    - 4.3. How is this relationship maintained?

**Organisational**

5. Who organises the use of the panel (who invites panellists for interviews, organises focus group etc.)?
    - 5.1. In what way will these costs be implemented? (users (researchers) or the administrator of the panel)

6. Is there a/who is the main manager of the panel?
    - 6.1. What do you call his/her job description?
    - 6.2. How was this person chosen?

6.3. What do his/her duties consist of?
6.4. How many FTEs does this cost?

7. What other functions are needed to keep this panel running?
7.1. What do the tasks consist of per function? (e.g. customer/panellist relations, cost/feedback results etc.)

8. Do you have partners/stakeholders and if so, who are they?
8.1. What does this partnership entail?
8.1.1.1. Do you exchange panellists?
8.1.1.2. Do you share results with each other?
8.1.1.3. Do you know about each other what research topics are being investigated? Do you coordinate with each other?
8.1.2. How are these relationships maintained?

**Communication / recruitment and engagement**

9. How do you recruit potential researchers for the panel?
9.1. How do you maintain these relationships?

10. How do you maintain relationships with panelists?
10.1 In what ways do you ensure transparency towards panellists?
10.2 How do you ensure that panelists keep coming back?
9.2.1. Is there feedback on the results of the surveys and if so, how?
10.3 Are there certain factors that contribute to panelists dropping out, and if so, what are they?
9.3.1. How large is the dropout rate?
9.3.2. In what ways can dropout be prevented? How do you deal with this?

**Economic**

11. What are the cost items? (e.g. fees, staff, ICT and travel costs)
11.1 Are there guidelines in terms of fees? Or based on own insights?
11.2. What are the sources of income?
11.2.1. Is it only by researchers using the panel? Or also government (ministry), donations etc.

12. In what way are panel members compensated?
12.1. Gift vouchers/points system? How does this work?
12.2. How do panellists perceive these reimbursements?

13. Does the management of the panel arrange the rewards? Or is this a task for the researchers?

14. Do panel members have to pay taxes on fees?

**Legal**

15. What legal frameworks do you have to adhere to?
15.1 Are there certain licences you need to have?

15.2. Which laws are important?

15.2.1. E.g. Privacy laws (AVG) (where is data kept and how is it handled?).

15.2.1.1. Can data from research A be reused for research B? How do you deal with this?

15.3 In what way and how often do you obtain permission/informed consent from panel members? (intellectual property)

15.3.1. Is this only at registration at the beginning or again at each survey?

15.4 Do you work with contracts/agreements with the panellists?

16. Are there any particular legal barriers to the project?

16.1 Do you have insight into what panel members think about how their data is handled? Are there people who do not participate due to privacy concerns?

**Operational / Inclusiveness / Recruitment**

17. How do you recruit new panellists?

17.1. How often do you recruit?

17.2. Is diversity of characteristics considered within the panel?

17.2.1. Are you recruiting specifically on this?

18. How do you select participants for the studies?

18.1. How do you ensure a diverse population?

19. How do you reach people for a study?

19.1. By mail? Do people know how to complete and return a questionnaire? Are participants trained in this?

20. How many times a year are they invited to participate in surveys?

21. Is there a limit to how long panel members can continue to participate? (e.g. due to learning curve)

22. How can researchers apply to use the panel for a study?

23. If you want to plan a date for e.g. a focus group, do you give panellists a choice or do you choose one date?

**Concluding with SWOT analysis**

24. What are/were the risks of setting up a panel?

25. What are/were the opportunities of setting up a panel?

26. What are/were the strengths/successes of this panel set up?

27. What are the weaknesses/fail factors of this panel set up?

Do you have any additions on this interview?

Thank the participant and end the interview.
