# Supplementary material for: Strategies for engaging “hard-to-reach” populations in a panel for digital health research: A qualitative study among experts
Source: PLOS Digit Health. 2025 Oct 9;4(10):e0001033. doi: 10.1371/journal.pdig.0001033 (PMC12510573; doi:10.1371/journal.pdig.0001033)
Supplement: S3 File — (PDF) [file pdig.0001033.s003.pdf]

Information for participation in research

Setting up a research panel with vulnerable citizens for research on digital health inclusiveness

Introduction

Dear reader,

With this information letter, we would like to ask you if you would like to participate in scientific research. Participation is voluntary. You are receiving this letter because, because of your expertise, you are being asked to participate in an interview as part of an ongoing study on setting up a panel on digital health.

You can read here what kind of research is involved, what it means for you and what is expected of you if you decide to participate.

Are you interested?

- Then read this letter carefully.
- Ask questions to the researcher who will give you this information.

Would you like to participate? Then fill in the form attached.

1. General information

The study is conducted by the Amsterdam UMC.

2. What is the purpose of the research?

The aim of the research is to look at the feasibility of setting up and maintaining a panel with vulnerable citizens for research on inclusiveness of digital care.

In a changing healthcare landscape where digital care is playing an increasing role, there is a growing emphasis on citizens' knowledge and skills to participate in it. Many digital care research projects have been criticised for targeting “easy sample” populations that are easy to reach and more likely to respond. This excludes groups that are less visible in society, such as citizens with low socioeconomic status (SES) and low health literacy (HL). One of the successful strategies proposed to increase participation of the latter groups is through panel surveys. For this reason, this study focuses on setting up and maintaining a panel with vulnerable citizens. In the interview, we will ask questions on topics such as: strategy, organisation, communication, economic, legal and operational feasibility, success and failure factors and ethical aspects (of other panels).

3. What happens if you participate in the survey?

This survey consists of filling in a questionnaire, which asks for your name, date of birth and profession. In addition, two researchers will conduct a one-time (digital) interview. Audio recordings of this will be made by means of a secure Smartphone app called Philips SpeechExec Enterprise Transcribe [v107.0.115.4].

4. What does participation mean for you?

There is no direct benefit to you yourself from participating in this study. Your participation may well contribute to gaining more knowledge on how to set up the above panel correctly. This may contribute to better inclusion of vulnerable populations in the future, leading to major health gains within these groups.

5. If you do not want to participate or want to quit the study.

Participation in the study is entirely voluntary. You may also decide to stop during the study. You do not have to say why you are stopping. However, you must inform the researcher immediately.
The data collected up to that point will still be used for the study.

6. What will we do with your data?

Are you participating in the study? Then you also give us permission to collect, use and store your data.

Why do we collect, use and store your data?

We collect, use and store your data to answer the questions in this survey. We want to publish the results of the survey.

What will we do with voice recordings?

During the study, we will make audio recordings of you. You will not be recognisable on these recordings. We will type out the sound recordings. After this, the recording itself will be destroyed.

How do we protect your privacy?

To protect your privacy, we give your data a code. On all your data, we put only this code. Data referring directly to you will then no longer be used. We keep the key to the code in a secure place in Amsterdam UMC. Only the researcher and members of the research team know which code you have. When we process or share your data, we always use only that code. In reports and publications about the research, no one can recall that it was about you.

How long do we keep your data?

We keep your data for 10 years at Amsterdam UMC.

Can you withdraw your consent to the use of your data?

You can withdraw your consent to the use of your data at any time. This applies to use in this study and to use in other research. But, do you withdraw your consent, and have researchers then already collected data for a study? Then they may still use this data.

Would you like to know more about your privacy?

Perhaps you would like to receive an electronic copy of data about you that was used for the study. This is possible. You can ask the researcher for this.
Would you like to know more about your rights when processing personal data? Then visit https://www.autoriteitpersoonsgegevens.nl/nl/over-privacy/persoonsgegevens
Do you have questions about your rights?

Or do you have a complaint about your privacy? If so, please contact the person responsible for processing your personal data, in this case: Corine Oldhoff-Nuijsink, [redacted] .

If you have complaints about your privacy, we recommend that you first discuss them with the research team. You can also go to the Data Protection Officer of Amsterdam UMC location AMC. Or you can file a complaint with the Personal Data Authority.

7. Will you receive compensation for participating?

If you participate in the study, we will reimburse any travel and parking costs you incur.

8. Do you have any questions?

This research has been reviewed by the non-WMO review committee of the Amsterdam UMC. According to this committee, this research does not fall under the Medical Research Involving Human Subjects Act (WMO). If you have any questions about this research, please contact the researcher: Corine Oldhoff-Nuijsink.

9. Do you have a complaint?

If you have a complaint, please discuss it with the researcher.
If you prefer not to, you can contact the Patient Service Care Support staff.
For location AMC:
- telephone number [redacted]
- e-mail address: [redacted]

Thank you for your attention.

Contact details:
Corine Oldhoff-Nuijsink
Researcher & Scientific lecturer Medical Information Science at Amsterdam UMC [redacted]
