# Supplementary material for: Strategies for engaging “hard-to-reach” populations in a panel for digital health research: A qualitative study among experts
Source: PLOS Digit Health. 2025 Oct 9;4(10):e0001033. doi: 10.1371/journal.pdig.0001033 (PMC12510573; doi:10.1371/journal.pdig.0001033)
Supplement: S4 File — (PDF) [file pdig.0001033.s004.pdf]

Annex: Participant consent form

Setting up a panel with vulnerable citizens for research on digital health inclusiveness

- I have read the information letter. I was also able to ask questions. My questions were sufficiently answered. I had enough time to decide whether to participate.

- I know that participating is voluntary. I also know that I can decide at any time not to participate after all or to stop the study. I do not have to give a reason for doing so.

- I consent to the collection and use of my data in the manner and for the purposes specified in the information letter.

- I give permission to keep my data for 10 years after this study [within the AMC].

- I want to participate in this study.

Please tick yes or no below:

- I consent to the collection and use of audio recordings. These recordings will be destroyed after being deregistered/end of study.

O yes

O no

- I give permission to be contacted again after this study for a follow-up study

O yes

O no

Name of test subject:_________________

Signature:

Date:_______________
